# Minimally invasive, robot-assisted reduction of grade 2 isthmic spondylolisthesis through posterior approach

**DOI:** 10.3171/2025.4.FOCVID2522

**Published:** 2025-07-01

**Authors:** Amanda N. Sacino, Gregory J. Cannarsa

**Affiliations:** 1 Apex Brain & Spine Neurosurgical Specialists, Naples; and; 2 Physicians Regional Collier Hospital Robotic Brain and Spine Neurosurgery Program, Naples, Florida

**Keywords:** isthmic spondylolisthesis, transforaminal lumbar interbody fusion, robotic-assisted surgery, cement augmentation of screws, minimally invasive spine surgery

## Abstract

Robotic assistance in spine surgery has provided new advancements for less invasive surgical options for patients who may otherwise not have qualified for these procedures in the past. The authors report the case of a 62-year-old woman with stage 4 metastatic breast cancer and osteoporosis who presented with debilitating bilateral lower extremity radiculopathy. MRI showed grade 2 spondylolisthesis at L5–S1 causing severe biforaminal stenosis. The video demonstrates use of the robot to ensure proper placement of the hardware as well as to preplan the L4 to S2-alar-iliac construct to allow for the least invasive surgery possible.

The video can be found here: https://stream.cadmore.media/r10.3171/2025.1.FOCVID24153

## Transcript

Here we present a case with the use of robotic assistance for the reduction of a grade 2 isthmic spondylolisthesis^1^ through a posterior approach.

### 0:32 Patient Background.

The patient is a 62-year-old female with a medical history significant for stage 4 metastatic breast cancer. She had undergone double mastectomy and radiation treatment, including to the lumbar spine. She is currently on immunotherapy and hormonal therapy. She also had a hysterectomy. She has osteoporosis that is currently being treated.

### 0:56 Presenting Symptoms and Initial Management.

The patient presented with low back pain and bilateral lower extremity radiculopathy that she rated as a 10 on the numeric rating scale. She had trialed conservative measures including physical therapy and epidural injections with no significant improvement. She had multiple hospitalizations for pain control and due to falls from left leg weakness when ambulating. Her physical exam was notable for diffuse weakness in the left leg secondary to pain.

### 1:26 Background Imaging.

CT and MRI scan of the lumbar spine showed bilateral pars defects at L5 and grade 2 spondylolisthesis at L5–S1 with severe degenerative endplate changes.

The arrow shows the left-sided severe foraminal stenosis correlating to the patient’s radiating leg pain.

### 1:52 Rationale for Procedure and Benefits of Robotic-Assisted Surgery.

Because the patient had prior abdominal surgery and radiation treatment, a posterior-only approach was recommended for reduction in the form of an L4 to S2-A-I fusion with cement augmentation of the L4 screws due to the poor bone quality from osteoporosis, bony metastasis, and prior radiation treatment.

Robotic-assisted surgery was beneficial in this case because it ensured the proper placement of hardware to avoid multiple passes in a patient with poor bone quality and because it allowed the surgeons to preplan the surgery to allow for the least invasive possible procedure in a patient who would not be able to tolerate an open procedure with high blood loss.^2–6^

### 2:37 Key Surgical Steps.

Key surgical steps are listed in this slide. The most important steps of the surgery include: use of the robotic navigation platform for hardware placement and to guide decompression, cement augmentation of the L4 screws to prevent pullout, and reduction of the spondylolisthesis via tandem tightening of the facet screws as well as sequential expansion of a temporary cage.

### 3:07 Surgical Procedure.

Once the patient has been positioned, intraoperative CT has been performed and loaded to the robot, operative planning of the fusion construct is performed. The position of each screw is assessed along with the overall alignment of the construct. The robot is then draped and brought into the field for incision planning. In the first stage of the surgery, the pedicles are then cannulated with navigated instruments in preparation for screw placement.^5^ Pedicle screws are then placed using robotic guidance to ensure accuracy with a single pass.^2,5^ The robotic navigation platform is then used to guide tubular retractor placement to assist with the next stage of the surgery.^3^ Microscopic decompression of the nerve roots and facetectomies utilizing robotic navigation is completed bilaterally.^3^ Reduction is then achieved by tightening facet screws sequentially from S2-A-I to L4 on the left-sided rod while expanding the interbody trial in tandem. The interbody cage is then placed using robotic navigation.^3^ Final x-rays are completed to show good placement of all hardware and reduction of the spondylolisthesis. Noted at L4 are the cement augmented pedicle screws to prevent pullout. The incision sizes ranged from 0.5 to 7 cm in length.

### 4:59 Postoperative Imaging.

Postoperative CT and MRI shows complete reduction of the spondylolisthesis at L5–S1 with good placement of the hardware and cement as well as significant improvement in lateral recess and foraminal stenosis.

### 5:21 Postprocedure Course.

The patient was able to continue with her immunotherapy and hormonal therapy in the perioperative period. Her numerical rating score for pain decreased from 10 to 4 immediately after surgery. She was able to ambulate independently within 12 hours of surgery and she was discharged from the hospital on postoperative day 2.

At 2 months after surgery, her pain score has decreased to 2 from her oncologic pain. Her preoperative radiculopathy has resolved.

## Disclosures

The authors report no conflict of interest concerning the materials or methods used in this study or the findings specified in this publication.

## Author Contributions

Primary surgeon: Sacino. Assistant surgeon: Cannarsa. Editing and drafting the video and abstract: both authors. Critically revising the work: both authors. Reviewed submitted version of the work: Sacino. Approved the final version of the work on behalf of both authors: Sacino.

## Supplemental Information

### Patient Informed Consent

The necessary patient informed consent was obtained in this study.

## References

[1] Alomari S Judy B Sacino AN Isthmic spondylolisthesis in adults. A review of the literature J Clin Neurosci 2022 101 124 130 35597059 10.1016/j.jocn.2022.04.042

[2] Sacino AN Materi J Davidar AD Robot-assisted atlantoaxial fixation: illustrative cases J Neurosurg Cases 2022 3 25 CASE22114 10.3171/CASE22114 PMC9210265 35733845

[3] Menta AK Weber-Levine C Jiang K Robotic assisted surgery for the treatment of spinal metastases: a case series Clin Neurol Neurosurg 2024 243 108393 38917745 10.1016/j.clineuro.2024.108393

[4] Davidar AD Jiang K Weber-Levine C Bhimreddy M Theodore N Advancements in robotic-assisted spine surgery Neurosurg Clin N Am 2024 35 2 263 272 38423742 10.1016/j.nec.2023.11.005

[5] Bhimreddy M Hersh AM Jiang K Accuracy of pedical screw placement using the ExcelsiusGPS robotic navigation platform: an analysis of 728 screws Int J Spine Surg 2024 18 6 712 720 39592194 10.14444/8660 PMC11687040

[6] Bhimreddy M Menta AK Fuleihan AA Beyond pedicle screw placement: future minimally invasive applications of robotics in spine surgery Neurosurgery 2025 96 3S S94 S102 39950789 10.1227/neu.0000000000003335

